# Understanding mammal avoidance of human settlements

**DOI:** 10.1111/1365-2656.70298

**Published:** 2026-07-01

**Authors:** Jonathan R. Potts, Luca Börger, Marlee A. Tucker, Federico Ossi, Scott W. Yanco, Diego Ellis‐Soto, Thomas Müller, Ruth Y. Oliver, Mario H. Alves, Walter Arnold, Nina Attias, Guillaume Bastille‐Rousseau, Jerrold L. Belant, J. David Blount, Dean E. Beyer, Francesca Cagnacci, Simon Chamaillé‐Jammes, Aung N. Chan, Eric K. Cole, Jessica S. Cornils, Rogerio Cunha de Paula, Vickie DeNicola, Arnaud L. J. Desbiez, Sarah R. Dewey, David Drake, Michael Egan, Jasper A. J. Eikelboom, Morgan Farmer, Mathieu Garel, Jacob R. Goheen, Hans Peter Hansen, Lars Haugaard, Mark Hebblewhite, Morten Heim, Miloš Ježek, Lilla Jordán, Douglas N. Kamaru, Miha Krofel, Tayler N. LaSharr, Peter Leimgruber, Anne Loison, Ryan A. Long, Matthias‐Claudio Loretto, Pascal Marchand, Erling Meisingset, Joerg Melzheimer, Kevin L. Monteith, John J. Morgan, Rasmus M. Mortensen, Rebekka Mueller, Atle Mysterud, Astrid Olejarz, Teresa Oliveira, Manuela Panzacchi, Ruben Portas, Hubert Potočnik, Herbert H. T. Prins, Laura R. Prugh, Nathan Ranc, Ralf Roeder, Christer M. Rolandsen, Çağan Şekercioğlu, Aldin Selimovic, Rachel Smiley, Erling Johan Solberg, Olav Strand, Peter Sunde, Carole Toïgo, Bram Van Moorter, Tana L. Verzuh, Bettina Wachter, Brittany L. Wagler, Jesse Whittington, Christopher C. Wilmers, George Wittemyer, Christian Rutz

**Affiliations:** ^1^ School of Mathematical and Physical Sciences, The University of Sheffield Sheffield UK; ^2^ Department of Biosciences & Centre for Biomathematics Swansea University Swansea UK; ^3^ Department of Environmental Science Radboud Institute for Biological and Environmental Sciences, Radboud University Nijmegen The Netherlands; ^4^ Animal Ecology Unit Research and Innovation Center (CRI), Fondazione Edmund Mach San Michele all'Adige Italy; ^5^ NBFC, National Biodiversity Future Center Palermo Italy; ^6^ School for Environment and Sustainability University of Michigan Ann Arbor Michigan USA; ^7^ Migratory Bird Center, Smithsonian's National Zoo and Conservation Biology Institute Washington District of Columbia USA; ^8^ Department of Environmental Science Policy and Management University of California Berkeley California USA; ^9^ Department of Ecology and Evolutionary Biology Yale University New Haven Connecticut USA; ^10^ Senckenberg Biodiversity and Climate Research Center (SBiK‐F) Frankfurt am Main Germany; ^11^ Department of Biological Sciences Goethe University Frankfurt Frankfurt am Main Germany; ^12^ Bren School of Environmental Science and Management, University of California Santa Barbara Santa Barbara California USA; ^13^ Department of Veterinary Medicine University of Bari Bari Italy; ^14^ Instituto de Conservação de Animais Silvestres (ICAS) Campo Grande Mato Grosso do Sul Brazil; ^15^ Department of Interdisciplinary Life Sciences, Research Institute of Wildlife Ecology University of Veterinary Medicine Vienna Vienna Austria; ^16^ Center for Wildlife Sustainability Research, Southern Illinois University Carbondale Illinois USA; ^17^ Department of Fisheries and Wildlife Michigan State University East Lansing Michigan USA; ^18^ School of Biological Sciences, University of Utah Salt Lake City Utah USA; ^19^ NBFC, National Biodiversity Future Centre Palermo Italy; ^20^ CEFE, CNRS, Univ Montpellier, EPHE, IRD Montpellier France; ^21^ Department of Zoology and Entomology Mammal Research Institute, University of Pretoria Pretoria South Africa; ^22^ Department of Fish, Wildlife and Conservation Biology Colorado State University Fort Collins Colorado USA; ^23^ Smithsonian National Zoo and Conservation Biology Institute, Conservation Ecology Center Front Royal Virginia USA; ^24^ U.S. Fish and Wildlife Service, National Elk Refuge Jackson Wyoming USA; ^25^ Centro Nacional de Pesquisa e Conservação de Mamíferos Carnívoros, Instituto Chico Mendes de Conservação da Biodiversidade Atibaia SP Brazil; ^26^ Field Engine Wildlife Research and Management Moodus Connecticut USA; ^27^ Royal Zoological Society of Scotland (RZSS) Edinburgh UK; ^28^ Instituto de Pesquisas Ecológicas (IPÊ) Nazaré Paulista São Paulo Brazil; ^29^ National Park Service, Grand Teton National Park Moose Wyoming USA; ^30^ Department of Forest and Wildlife Ecology University of Wisconsin‐Madison Madison Wisconsin USA; ^31^ Center for Wildife Sustainability Research, Southern Illinois University Carbondale Illinois USA; ^32^ Laboratory of Geo‐information Science and Remote Sensing, Wageningen University and Research Wageningen The Netherlands; ^33^ Department of Forest and Wildlife Ecology University of Wisconsin Madison Wisconsin USA; ^34^ Office Français de la Biodiversité, Service Anthropisation et Fonctionnement des Ecosystèmes Terrestres Gières France; ^35^ Department of Natural Resource Ecology & Management Iowa State University Ames Iowa USA; ^36^ Global Resource Systems, Iowa State University Ames Iowa USA; ^37^ Department of Ecoscience Aarhus University Aarhus Denmark; ^38^ Wildlife Biology Program, Franke College of Forestry and Conservation, University of Montana Missoula Montana USA; ^39^ Terrestrial Ecology Department Norwegian Institute for Nature Research Trondheim Norway; ^40^ Department of Game Management and Wildlife Biology, Faculty of Forestry and Wood Sciences Czech University of Life Sciences Suchdol Czech Republic; ^41^ Department of Evolutionary Ecology Leibniz Institute for Zoo and Wildlife Research Berlin Germany; ^42^ Department of Wildlife Diseases Leibniz Institute for Zoo and Wildlife Research Berlin Germany; ^43^ Biotechnical Faculty University of Ljubljana Ljubljana Slovenia; ^44^ Department of Zoology and Physiology, Haub School of Environment and Natural Resources, Wyoming Cooperative Fish and Wildlife Research Unit University of Wyoming Laramie Wyoming USA; ^45^ Smithsonian National Zoo and Conservation Biology Institute Front Royal Virginia USA; ^46^ Laboratoire d'Écologie Alpine, UMR UGA‐USMB‐CNRS 5553, Université de Savoie Mont‐Blanc Le Bourget‐du‐Lac France; ^47^ Department of Fish and Wildlife Sciences University of Idaho Moscow Idaho USA; ^48^ Office Français de la Biodiversité, Service Anthropisation et Fonctionnement des Ecosystèmes Terrestres Pérols France; ^49^ Department of Forestry and Forestry Resources Norwegian Institute of Bioeconomy Research Tingvoll Norway; ^50^ Environmental Studies Department University of California Santa Cruz California USA; ^51^ Department of Biosciences, Centre for Ecological and Evolutionary Synthesis University of Oslo Oslo Norway; ^52^ Department of Nature, Land and Society Norwegian Institute for Nature Research Oslo Norway; ^53^ Department of Biology, Biotechnical Faculty University of Ljubljana Ljubljana Slovenia; ^54^ Department of Animal Sciences Wageningen University and Research Wageningen The Netherlands; ^55^ School of Environmental and Forest Sciences, University of Washington Seattle Washington USA; ^56^ Université de Toulouse, INRAE, CEFS Castanet‐Tolosan France; ^57^ Department of Molecular Biology and Genetics Koç University Istanbul Türkiye; ^58^ Parks Canada Agency, Banff National Park Resource Conservation Banff Alberta Canada; ^59^ Environmental Studies Department Universtiy of California Santa Cruz California USA; ^60^ Centre for Biological Diversity, School of Biology, University of St Andrews St Andrews UK

**Keywords:** anthropause, bio‐logging, GPS tracking, human–wildlife coexistence, step selection, sustainability

## Abstract

1. Anthropogenic land conversion is putting increasing pressure on wildlife populations around the world. To mitigate impacts, it is necessary to develop a detailed mechanistic understanding of how animals are affected by different types of human activity. A key challenge is to disentangle the effects of static infrastructure, like roads or buildings, and the presence of humans in the landscape.
2. To address this question, we examined if terrestrial mammals altered their movement behaviour around buildings in response to reduced human mobility during COVID‐19 lockdowns. We compiled GPS tracking data from 35 study sites across five continents, for 10 carnivore species and 13 herbivore species, totalling >1 million location records from 586 individuals. For each study, we used integrated step selection analysis to test the extent to which animals changed their avoidance of buildings as lockdown took effect, leveraging the recently released Microsoft MLBuildings dataset of global building locations.
3. Analysis of population‐level effects revealed that, in areas with high Human Footprint Index (HFI), animals tended to show a significant reduction in their avoidance of buildings during lockdown, but not in low HFI areas. No such trend was detected during equivalent periods in years other than 2020, indicating that behavioural changes were a result of reduced human mobility during lockdowns.
4. Overall, our findings suggest that animals living alongside humans exhibit greater plasticity when people change their behaviour, likely indicating the combined effects of environmental filtering and habituation. More generally, our study provides a critical first step towards developing evidence‐based tools for forecasting how wildlife movement behaviour may change in response to different land‐use strategies, human activities, conservation interventions or environmental perturbations.

## Graphical abstract

Terrestrial mammals often avoid human settlements, but is this a result of human activity around buildings or the built infrastructure per se? Using data on animal movement before and during the COVID‐19 lockdowns, this paper disentangles these effects, showing how changes in human mobility affect animals' avoidance of buildings.

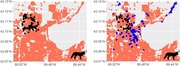

## 1 INTRODUCTION

Land conversion forces humans and animals into close contact on a planetary scale (Ma et al., 2024; Nyhus, 2016). Mitigating impacts on wildlife requires a deep mechanistic understanding of the effects of human activity on animal behaviour (Ellis‐Soto et al., 2023; Gomez et al., 2025). The survival of individual animals, and ultimately entire populations, depends on their ability to move efficiently across the landscape, to access key resources, like food, shelter and breeding sites (Abrahms et al., 2021; Nathan et al., 2008). Yet movement can be compromised by both land modification (fragmented habitat, roads, fences, buildings and other infrastructure) and human mobility (the movement of people and their vehicles) (Doherty et al., 2021; Ellis‐Soto et al., 2023; Meekan et al., 2017; Rutz, 2022b; Tucker et al., 2018). Disentangling the effects of these static and dynamic components of human activity is crucial for mechanistic interpretation, but challenging, since they are usually confounded, and experimental manipulations are impossible to achieve at scale (Gomez et al., 2025; Rutz et al., 2020).

With urban expansion projected to surge over the coming decades (Angel et al., 2011; Huang et al., 2019; Seto et al., 2012), investigating the impact of human settlements on wildlife movement is of particular importance. Vertebrate biodiversity declines steeply along gradients of urbanisation (McKinney, 2008; Newbold et al., 2015), but it remains unclear—for the reasons outlined above—if wildlife predominantly struggles with the built environment itself or the presence of humans and moving vehicles. Although under tragic circumstances, the COVID‐19 lockdowns of early 2020 provided an unprecedented opportunity to address this long‐standing question (Rutz, 2022b; Rutz et al., 2020). In many areas, government restrictions caused a sudden, short‐term reduction in human mobility levels, while the distribution of buildings, roads and other infrastructure remained effectively unchanged—dissociating key stressors in a quasi‐experimental manner (Bates et al., 2020; Rutz, 2022b; Rutz et al., 2020).

A growing body of research documents lockdown impacts on the natural world (Bates et al., 2021; Gill et al., 2024; Tucker et al., 2023). While some species seem to have benefited from reduced levels of human mobility, others have come under increased pressure (e.g. because of reductions in anthropogenic food sources), and in many cases, no effects were detectable (Bates et al., 2021; Mikula et al., 2024). Two studies conducted pioneering global‐scale assessments of potential changes in the movement behaviour and activity of terrestrial mammals. Analyses of GPS tracking data for 2300 tagged individuals revealed that animals tended to venture closer to roads during lockdowns in areas of high human footprint (compared to 2019), and moved larger distances in areas experiencing particularly strict lockdowns (Tucker et al., 2023). Camera trapping data from 102 projects worldwide provided critical context for these findings, demonstrating that changes in the amount and timing of animals' activity varied across taxa and environmental contexts (Burton et al., 2024).

Despite these important advances, there have been few attempts to model lockdown‐related changes in wildlife movement behaviour in finer detail, going beyond basic movement metrics or occurrence detection (Oliver et al., 2026; Patchett et al., 2025). Here, we help fill this critical knowledge gap with a detailed analysis of whether terrestrial mammals changed their avoidance of buildings during lockdowns, compared to baseline years. We collated 35 GPS tracking datasets for 10 carnivore and 13 herbivore species across five continents (Table 1), covering a wide range of environmental contexts—from highly built‐up areas, such as New York City, to habitats with very few buildings, such as parts of rural Norway. To explore further the context dependence in behaviour observed in earlier studies, we investigated whether a lockdown effect on the avoidance of buildings might differ between more and less anthropogenically impacted areas, as measured by the Human Footprint Index (HFI; Venter et al., 2016; Williams, Venter, et al., 2020).

**TABLE 1:** Summary of the datasets used.

ID	Location	Species	Inds	Fixes	Fix int	LD start	LD end	Cont	Diet	HFI
NR	Norway	Reindeer (*Rangifer tarandus*)	29	28,478	3 h	13 March	30 May	None	H	3.31
NC	Namibia	Cheetah (*Acinonyx jubatus*)	2	6824	15 mins	28 March	4 May	2019	C	5.63
FC	France	Chamois (*Rupicapra rupicapra*)	4	3504	2 h	17 March	11 May	None	H	6.18
WB	Washington, US	Bobcat (*Lynx rufus*)	20	9078	4 h	23 March	31 May	2019	C	7.68
ZW	Czech Republic	Grey wolf (*Canis lupus*)	1	650	4.5 h	16 March	16 May	2021	C	7.75
WS	Wyoming, US	Bighorn sheep (*Ovis canadensis*)	63	152,314	1 h	13 March	14 May	None	H	8.00
KL	Kenya	Lion (*Panthera leo*)	5	684	2 h	1 March	16 May	2019	C	8.02
MW	Michigan, US	Grey wolf (*Canis lupus*)	2	2380	2 h	25 March	11 June	None	C	8.66
ZD	Czech Republic	Red deer (*Cervus elaphus*)	5	22,498	1 h	16 March	16 May	2019	H	8.74
YE	Myanmar	Asian elephant (*Elephas maximus*)	9	23,769	2 h	16 March	31 May	None	H	8.78
SE	South Africa	African elephant (*Loxodonta africana*)	8	96,088	12 mins	27 March	13 May	2019	H	8.85
CW	Canada	Grey wolf (*Canis lupus*)	4	4900	2 h	17 March	31 May	2019	C	9.45
SW	South Africa	Blue wildebeest (*Connochaetes taurinus*)	8	23,983	1 h	22 March	13 May	None	H	10.5
WY	Washington, US	Coyote (*Canis latrans*)	12	9870	4 h	23 March	31 May	2019	C	10.8
WD	Wyoming, US	Mule deer (*Odocoileus hemionus*)	133	250,591	1 h	13 March	14 May	2019	H	11.3
SZ	South Africa	Plains zebra (*Equus quagga*)	5	16,967	1 h	22 March	31 May	None	H	11.7
SL	Slovenia	Eurasian lynx (*Lynx lynx*)	3	811	3 per day	15 March	16 May	2021	C	12.0
ME	Mozambique	African elephant (*Loxodonta africana*)	6	77,063	15 mins	1 April	16 May	None	H	12.7
NM	Norway	Moose (*Alces alces*)	102	102,324	3 h	13 March	30 May	2019	H	14.4
ND	Norway	Red deer (*Cervus elaphus*)	18	31,837	1 h	13 March	30 May	2019	H	14.4
TL	Turkey	Eurasian lynx (*Lynx lynx*)	3	1047	6 h	16 March	15 May	None	C	14.4
AD	Austria	Red deer (*Cervus elaphus*)	5	1250	8.5 h	16 March	20 April	2021	H	14.8
TW	Turkey	Grey wolf (*Canis lupus*)	2	918	5 h	16 March	15 May	2019	C	15.1
BA	Brazil	Giant anteater (*Myrmecophaga tridactyla*)	8	2875	1 h	24 March	11 May	None	C	15.5
DD	Denmark	Red deer (*Cervus elaphus*)	7	27,881	1 h	13 March	18 May	2021	H	16.2
WE	Wyoming, US	Elk (*Cervus canadensis*)	35	58,283	90 mins	13 March	14 May	2019	H	16.8
ID	Illinois	White‐tailed deer (*Odocoileus virginianus*)	37	37,098	2 h	21 March	30 May	2021	H	17.8
WC	Wisconsin, US	Coyote (*Canis latrans*)	1	352	3 h	13 March	25 May	2019	C	18.3
SJ	Slovenia	Golden jackal (*Canis aureus*)	4	3493	2 h	15 March	16 May	None	C	18.4
CP	California, US	Puma (*Puma concolor*)	7	4717	4 h	17 March	16 May	2019	C	18.9
BW	Brazil	Maned wolf (*Chrysocyon brachyurus*)	2	2834	1 h	24 March	6 May	2019	C	19.6
CD	Italy	Roe deer (*Capreolus capreolus*)	10	18,829	1 h	10 March	3 May	2019	H	22.3
PD	Pennsylvania, US	White‐tailed deer (*Odocoileus virginianus*)	17	14,293	4 h	16 March	28 May	2019	H	29.7
NY	New York, US	White‐tailed deer (*Odocoileus virginianus*)	6	68,304	15 mins	23 March	8 June	2018	H	34.3
WF	Wisconsin, US	Red fox (*Vulpes vulpes*)	3	535	1 h	13 March	25 May	2022	C	40.7
		Totals	586	1,107,322						

*Note*: In turn, the columns show the study identifier (ID), country (or if in the USA, the state) where the majority of fixes were recorded (Location), study species (Species), number of tagged individuals used (Inds), number of fixes used (Fixes), time interval between successive fixes (Fix int), lockdown (LD) start, LD end, control year (Cont), Diet (C for carnivore and H for herbivore), and human footprint index (HFI). Rows are ordered by increasing values of HFI. Data are found on Zenodo, https://doi.org/10.5281/zenodo.15309885.

The key metric in our analyses is the response of tagged animals to the presence of buildings in an area, before and during lockdowns. While infrastructure encompasses other elements—like roads, fences or windfarms—buildings provided a clear, biologically relevant, globally mapped variable for disentangling key drivers of animal movement behaviour in human‐modified landscapes. They can be viewed as providing a proxy for likely human presence, offering a complementary perspective to earlier analyses of road effects (Tucker et al., 2023). We hypothesised that animals would alter their movement response towards buildings during lockdowns, if they are sensitive to changes in human mobility levels (specifically, less avoidance), but show no change otherwise.

Our finding that wildlife movement behaviour is sensitive to temporal changes in human mobility levels has implications going well beyond understanding lockdowns' impacts. Importantly, our results can be used to parameterise process‐based movement models that are capable of forecasting wildlife responses to different land‐use and conservation scenarios (Gomez et al., 2025; Verzuh et al., 2026)—arguably one of the biggest challenges in developing robust strategies for fostering sustainable human–wildlife coexistence (Carter & Linnell, 2023; Riotte‐Lambert & Matthiopoulos, 2020).

## 2 METHODS

Our study approach was comprised of three consecutive steps. First, we assembled datasets from various studies containing GPS locations of terrestrial mammals that had been tracked both prior to, and during, the COVID‐19 lockdowns in their respective countries. Second, we analysed each study separately to examine whether animals changed their movement response to buildings during lockdown as compared with prior to lockdown, using integrated step selection analysis (iSSA). Third, we performed a meta‐analysis of the individual iSSA results to test whether there was a trend in those responses and how that trend depended upon the extent of anthropogenic impact in the study area, as measured by the HFI. We describe each step in detail below.

### 2.1 Protocol for obtaining datasets

This study is a subproject of the COVID‐19 Bio‐Logging Initiative (henceforth ‘the Initiative’)—a global research consortium investigating the impact of pandemic lockdowns on animal movement behaviour (Rutz et al., 2020). For our analysis, we decided to focus on the lockdowns in the first half of 2020, as these were typically the most stringent in each country, and were put into effect in almost all countries in the world, although the precise timings did vary. We advertised our project through the mailing list for Initiative partners (>600 recipients) and also contacted all data providers on a previous Initiative paper (Tucker et al., 2023), with a few exceptions where we knew the data were not open access. We analysed almost all datasets that were provided to us which (a) came with assurances that they can be published open access and (b) had GPS data on animals tracked through the start of lockdown. As long as (a) and (b) were met, we only turned down data a priori where either the animals were in hibernation during the start of lockdown (e.g. brown bears in Serbia and Poland), so did not move during the pre‐lockdown period, or they lived in an area where there are no buildings (e.g. a reserve or an uninhabited island). On analysing the data, we were forced to exclude 10 further datasets: six because we were unable to extract building data from MLBuildings for the locations where the animals were observed, and four because the animals did not approach buildings closely enough to perform iSSA both before and during the lockdowns (i.e. moving within the same 100m×100m square as a building: see Section 2.2). After this sifting, we were left with 35 datasets (Table 1). Details about ethical approvals and other research licences are provided in Supporting Information Appendix F and Table S1.

### 2.2 Per‐study analysis: iSSA

**FIGURE 1:**
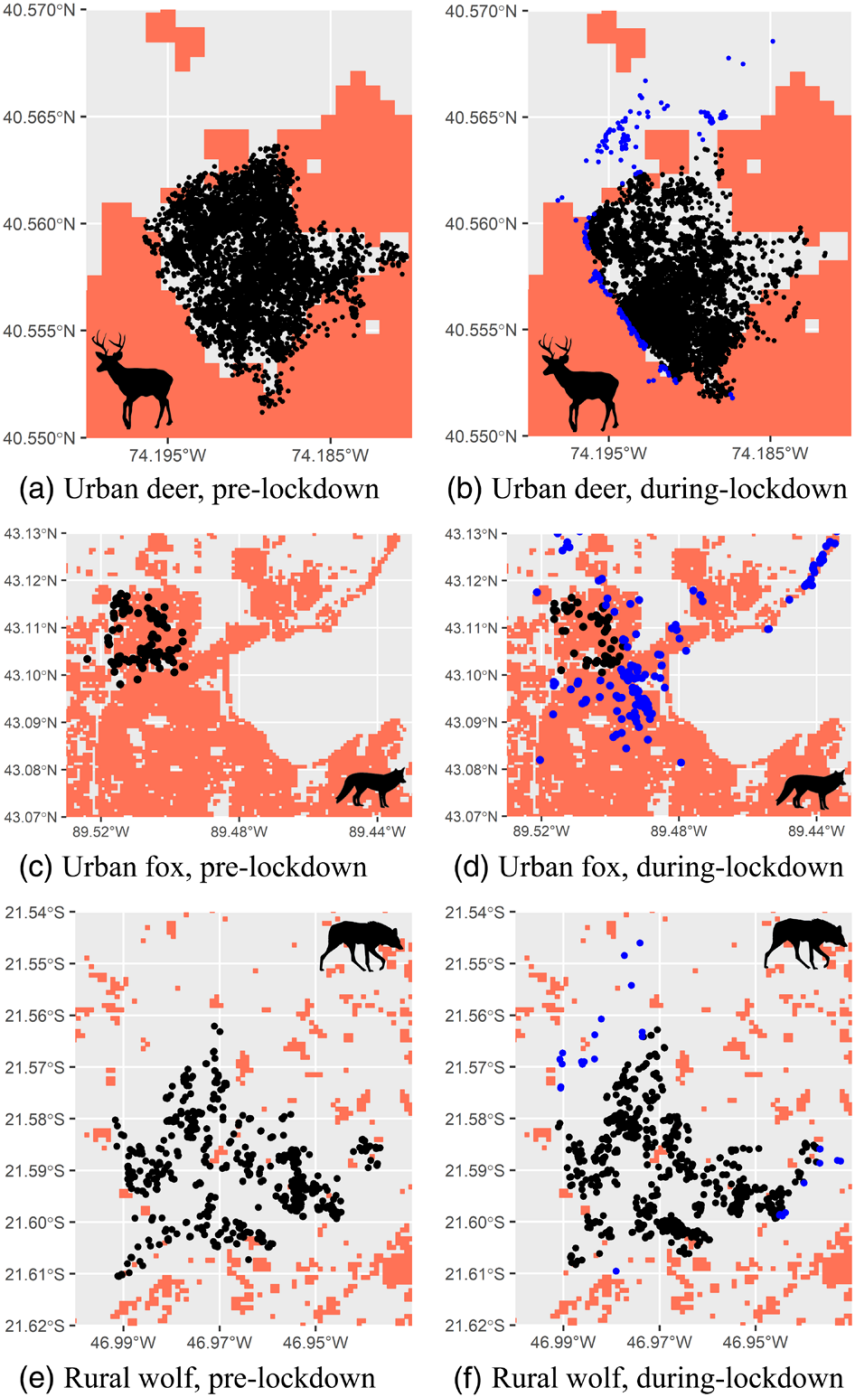
Examples of animal locations pre‐ and during‐lockdown. Dots show animal locations, which are in black if they are within the pre‐lockdown range and blue if outside that range (measured as the minimum convex polygon of location fixes). Red squares are 100m×100m cells containing buildings. Panel (a) shows a deer in New York, USA (study NY, Table 1) that spent much of its time pre‐lockdown well within a park area in‐between surrounding buildings. During lockdown (b), it spent a lot of time close to the buildings in the south‐west, as well as exploring over a greater area to the north. Panels (c) and (d) show a red fox in Madison, USA, (study WF) that increased the area over which it roamed during the lockdown, leading to more encounters with buildings in this city location (the large area with no buildings in the eastern half of the plot is Lake Mendota). In more rural locations, the effect of lockdown is sometimes not so easy to see visually (e, f), but we nonetheless observed a statistically significant lockdown effect for this maned wolf living just outside of Mococa, Brazil (study BW).

For each of the 35 studies, we identified the most common time interval between fixes in each dataset (detailed in Table 1) and then removed any location where the two step‐times prior to the location were each not approximately equal to (within 50% of) this time interval. (Nb. two consecutive steps of approximately the same time interval are required to account for turning angles.) We split each resulting track into a *pre‐lockdown* track, consisting of all locations recorded between 1st January 2020 and the day before lockdown, and a *during‐lockdown* track, consisting of all locations recorded during the lockdown period (see Table 1 for lockdown dates for each study). This yielded 70 datasets, 35 pre‐lockdown and 35 during lockdown, each containing a set of contiguous tracks for the individuals in the study. Examples of location data pre‐ and during‐lockdown are shown in Figure 1, to help visualise how these animals altered their ranges in response to lockdowns.

Each dataset was analysed separately using integrated step selection analysis (iSSA; Avgar et al., 2016). The explanatory variable of interest for our study is the presence or absence of buildings. To measure this, we split each study area into a 100m×100m square grid, and used Microsoft's ‘Global ML Building Footprints’ database to ascertain whether or not each square contained a building. The resulting variable has a value of $Z_{\text{build},i}\left(x,y\right)=1$ if the location $\left(x,y\right)$ is in a square containing at least one building and 0 otherwise, where i indexes the 35 studies. The coefficient of $Z_{\text{build},i}\left(x,y\right)$ in the step selection function is denoted $\beta_{\text{build},i}$, giving the size of the attraction (if positive) or avoidance (if negative) effect.

We chose a 100m resolution as it is sufficiently large so that small gaps between buildings (such as residential gardens or roads) count as part of the human settlement that animals respond to, but not so large as to encompass a lot of non‐building area around a settlement. We also tested the effect of changing that resolution (Supporting Information Appendix A). For the purposes of our study, single isolated buildings are counted as a ‘settlement’, alongside areas such as villages, towns, and cities. While not ideal, the alternative would be to deliberately demarcate buildings that are isolated and ignore them in the study. This would introduce an arbitrary cut‐off, however, whereby a small collection of >1 buildings would count as a settlement but a single building would not; nor would a building that is just a few hundred metres outside a village count as part of the village settlement. Therefore, we concluded that the most justifiable choice was to define any 100m square containing a building as part of a settlement.

In addition to building presence, $Z_{\text{build},i}$, we controlled for various other variables that are likely to affect movement of many terrestrial mammals: (a) presence or absence of forest, downloaded from the European Commission's ‘Global Forest Cover 2020’ dataset (Bourgoin et al., 2023); (b) distance to the central point of the data for each individual, to model any intrinsic home ranging tendency; (c) elevation above sea level, extracted from the ‘NASA SRTM Digital Elevation 30m’ dataset (Farr et al., 2007); (d) cosine of the turning angle, to model any directional autocorrelation between steps; and (e) step length, to correct for any error in the estimation of intrinsic step length (Forester et al., 2009). The iSSA model we used to analyse each of our datasets had $Z_{\text{build},i}$ included as an individual‐level random effect, with all control variables (a–e) as fixed effects (except for a few minor alterations in some datasets due to data restrictions; see Supporting Information Appendix E). We also repeated our analysis without controlling for covariates (a–c) to check whether our results are robust to removing the control variables (Supporting Information Appendix C).

Our iSSA model was fitted using the ‘Poisson trick’ of Muff et al. (2020) and the glmmTMB package in R. Each observed location was paired with 10 control locations. Control locations were calculated by drawing a step length from an exponential distribution with a mean derived from the empirical step lengths, and bearing drawn from a uniform distribution (both of which were corrected for as part of the iSSA procedure: see (d) and (e) above and Avgar et al. (2016)).

### 2.3 Meta‐analysis of iSSA results

To understand the overall effect of buildings on animal movement across all study systems, we performed meta‐analyses of the estimated $\beta_{\text{build},i}$‐values (mean and standard deviations) arising from our per‐study step selection analyses. For each of the pre‐lockdown effect sizes ($\beta_{\text{build},i}^{\mathrm{pre}}$), during‐lockdown effect sizes ($\beta_{\text{build},i}^{\mathrm{dur}}$) and the difference between them ($\beta_{\text{build},i}^{\text{diff}}=\beta_{\text{build},i}^{\mathrm{dur}}-\beta_{\text{build},i}^{\mathrm{pre}}$), we fitted two models. First, to assess the mean avoidance of buildings across the studies, we fitted a model of the form $\beta_{\text{build},i}^{X}=\mu^{X}+\epsilon_{i}$ for each $X\in \left\{\mathrm{pre},\mathrm{dur},\text{diff}\right\}$, where $\epsilon_{1},\ldots ,\epsilon_{35}$ are i.i.d. normal random variables. Here, $\mu^{\mathrm{pre}}$ (resp. $\mu^{\mathrm{dur}}$, $\mu^{\text{diff}}$) is the mean avoidance across all the studies in the pre‐lockdown period (resp. during lockdown period, difference between the two). The purpose of this test is first to identify whether there is a consistent response to buildings across studies pre‐ and during‐lockdown (respectively, $\mu^{\mathrm{pre}}$ and $\mu^{\mathrm{dur}}$ are significantly different from zero); then to see whether there was a significant difference between the pre‐ and during‐lockdown responses to buildings, by testing whether $\mu^{\text{diff}}$ is significantly different to zero.

Second, to assess the extent to which the effect size depends upon the HFI, we fitted a model of the form $\beta_{\text{build},i}^{X}=\alpha_{0}^{X}+\alpha_{1}^{X}H_{i}+\epsilon_{i}$, where $H_{i}$ is the HFI of the area used by the animals in study i.

A negative (resp. positive) value of $\alpha_{1}^{X}$ means that the extent of the increase in avoidance of (resp. attraction to) buildings during lockdown (as compared with pre‐lockdown) increases with HFI. The parameter $\alpha_{0}^{X}$ is an intercept (i.e. the expected value of $\beta_{\text{build},i}^{X}$ if HFI is zero). Per‐study HFI values (see Table 1) were calculated by averaging the HFI across all fixes and control locations, to obtain an estimate of the average HFI in the area available to the animal.

Meta‐analyses were performed using the R package metafor (Viechtbauer, 2010). This performs regression analysis, while taking into account the different standard errors arising from each study in a statistically consistent way. Those studies with large uncertainty in the $\beta_{\text{build},i}$‐values are given less weight. This could arise, for example, due to smaller sample sizes in some studies, so this takes account of the variation in the numbers of both individuals and fixes across our datasets (Table 1). As another example, larger standard errors could also occur in studies with fewer locations close to buildings.

We also assessed the effect of lockdown stringency, using an analogous approach to the linear model with an HFI slope discussed above (i.e. $\beta_{\text{build},i}^{X}=\alpha_{0}^{X}+\alpha_{1}^{X}H_{i}+\epsilon_{i}$) but with $H_{i}$ replaced with a measure of lockdown stringency. Details of this test (methods and results) are given in Supporting Information Appendix D.

Additionally, we checked for any effect of phylogenetic relatedness on our results and also controlled for the differences in frequency of fixes between studies, with methods and results detailed in Supporting Information Appendix C.

To check that our observations were driven by lockdowns and not by seasonal changes, we compared our analysis with an identical analysis of a control year—that is a year other than 2020. By default, we used 2019 data, but for some datasets we did not have sufficient 2019 data so used alternative control years, and for others, we had no usable control year (see Table 1 and Supporting Information Appendix B). We performed identical analysis for the control years as for the 2020 data, and denote by $\beta_{N,i}^{\text{contX}}$ the control year β‐value analogous to $\beta_{N,i}^{X}$. We also calculated $\beta_{\text{build},i}^{\mathrm{dur}}-\beta_{\text{build},i}^{\text{contdur}}$, the difference between the effect size in 2020 and the control year, and fitted identically structured models to the rest of our analysis—that is both random effects ($\beta_{\text{build},i}^{\mathrm{dur}}-\beta_{\text{build},i}^{\text{contdur}}=\mu^{\text{contdur}}+\epsilon_{i}$) and mixed effects ($\beta_{\text{build},i}^{\mathrm{dur}}-\beta_{\text{build},i}^{\text{contdur}}=\alpha_{0}^{\text{contdur}}+\alpha_{1}^{\text{contdur}}H_{i}+\epsilon_{i}$) – to see directly if animals were behaving differently during lockdown as compared with the same period in the control years. Since only 24 of the 35 datasets had sufficient data to calculate this value (Supporting Information Appendix B), we also re‐calculated $\alpha_{1}^{\text{diff}}$ for the 2020 data, but restricting our analysis to the 24 datasets we used for the control years. This allowed for a like‐for‐like comparison, albeit with a smaller sample of studies.

## 3 RESULTS

**FIGURE 2:**
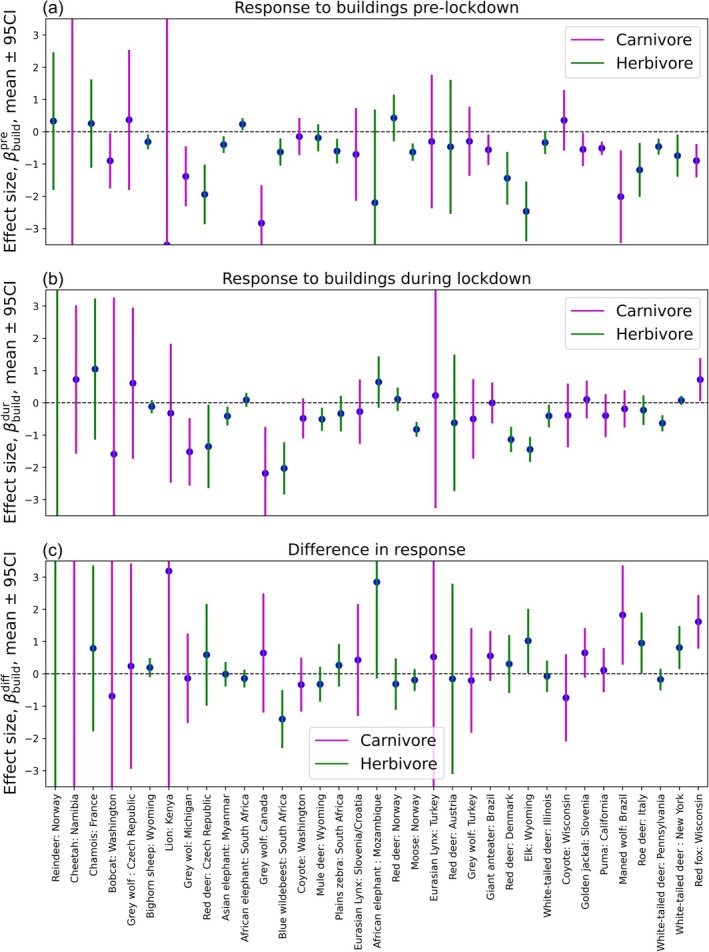
Per‐study effect of response to buildings. Panels (a) and (b) display the pre‐lockdown and during‐lockdown effect sizes, respectively. Positive values indicate an attraction to areas where there are buildings, negative values an avoidance. (c) The response strength during lockdown minus pre‐lockdown, that is $\beta_{\text{build},i}^{\text{diff}}=\beta_{\text{build},i}^{\mathrm{dur}}-\beta_{\text{build},i}^{\mathrm{pre}}$. Positive values indicate greater avoidance pre‐lockdown compared to during lockdown (negative values indicate less avoidance). In both panels, the studies are listed from left to right in order of increasing Human Footprint Index. Green bars are for herbivores and magenta for carnivores; blue dots are mean values. Note that for some studies the confidence intervals are too large to fit within the panels. Also, a few of the mean values (blue dots) do not fit within the panels. For all cases, numerical values can be found in Table S3.

Prior to lockdown, the general tendency was for animals to avoid buildings, with 20 of our 35 studies showing a significant avoidance effect (Figure 2a). In contrast, during lockdown, only 11 of 35 studies exhibited significant avoidance (Figure 2b), further supported by the difference between the during‐ and pre‐lockdown effects 2c, denoted $\beta_{\text{build},i}^{\text{diff}}=\beta_{\text{build},i}^{\mathrm{dur}}-\beta_{\text{build},i}^{\mathrm{pre}}$ for each study i, listed in order of ascending HFI.

For the simple model that does not account for HFI, namely $\beta_{\text{build},i}^{X}=\mu^{X}+\epsilon_{i}$, the avoidance of buildings was significant both pre‐lockdown, where $\mu^{\mathrm{pre}}=-0.64\pm 0.23$ (mean ± 95% CI) (p<0.0001), and during lockdown, where $\mu^{\mathrm{dur}}=-0.41\pm 0.22$ (p=0.0003). While this indicates a mean effect size reduction of about 36%, it turns out that this reduction is not statistically significant (specifically, $\mu^{\text{diff}}=0.17\pm 0.23$, p=0.13) so there is no evidence for a lockdown effect when not accounting for HFI.

**FIGURE 3:**
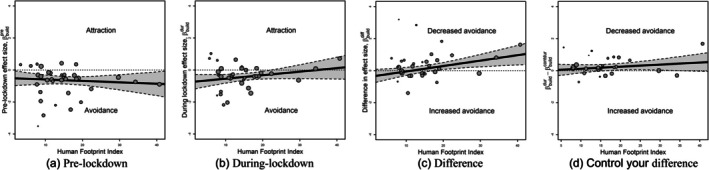
Response to buildings as a function of Human Footprint Index (HFI). (a) Prior to lockdown, while animals generally tended to show avoidance of buildings (negative values), there was no significant correlation between HFI and building avoidance. (b) During lockdown, this changed, so that animals in high‐HFI areas showed lower avoidance (a less negative effect size). The difference is shown in (c), while (d) plots the same measurement but for the control years. The size of the bubbles denotes their weight in the model (determined by the standard error of the respective β‐value), the black line is the best fit line, and the grey shaded area is the 95% confidence region.

However, when assessing the extent to which the change in effect size depends upon HFI, the lockdown effect was much clearer. Pre‐lockdown, we observed a non‐significant relationship between avoidance of buildings and HFI ($-\alpha_{1}^{\mathrm{pre}}=0.0089\pm 0.029$, p=0.54, Figure 3a; note that avoidance of buildings is encoded in $-\alpha_{1}^{\mathrm{pre}}$, so negative relationships slope upwards rather than downwards). During lockdown, this relationship switched to a negative relationship ($-\alpha_{1}^{\mathrm{dur}}=-0.022\pm 0.027$), albeit not significant at 5% (p=0.11, Figure 3b). Importantly, though, the difference between pre‐ and during‐lockdown was both significant and negative ($-\alpha_{1}^{\text{diff}}=-0.034\pm 0.024$, p=0.0060, Figure 3c). This means that the reduction in avoidance during lockdown was much more marked in high‐HFI areas compared with low‐HFI areas. The mean effect size was about twice as large for carnivores ($-\alpha_{1}^{\text{diff}}=-0.054\pm 0.032$, p=0.0008) as for herbivores ($-\alpha_{1}^{\text{diff}}=-0.020\pm 0.029$, p=0.19), although as p=0.11 for the difference in effect sizes, this result should be treated with caution.

For the control years, $\alpha_{1}^{\text{diff}}$ was not significantly different from zero ($-\alpha_{1}^{\text{diff}}=-0.014\pm 0.022$ p=0.22, Figure 3d), suggesting this trend in $\alpha_{1}^{\text{diff}}$ is a result of lockdowns rather than seasonality. When re‐calculating $\alpha_{1}^{\text{diff}}$ for the 2020 data, but restricting our analysis to the 24 datasets used for the control years, we obtained a value of $-\alpha_{1}^{\text{diff}}=-0.032\pm 0.025\left(p=0.015\right)$, which is not significantly different (p=0.93) from the $\alpha_{1}^{\text{diff}}$‐value (of $-\alpha_{1}^{\text{diff}}=-0.034\pm 0.024$) calculated with all 35 datasets. These results combine to suggest that the observed trend in $\alpha_{1}^{\text{diff}}$ from 2020 is likely due to lockdowns, but we cannot completely rule‐out seasonal effects.

When calculating $\beta_{\text{build},i}^{\mathrm{dur}}-\beta_{\text{build},i}^{\text{contdur}}$, the difference in the avoidance effect between the during‐lockdown period and the same period of year from the control years, we found the avoidance effect to be significantly higher in the control years, as one would expect if lockdowns were causing animals to avoid buildings less ($\mu^{\text{contdur}}=0.20\pm 0.18$, p=0.030). That said, we did not find a significant relationship between HFI and the difference between effect sizes in 2020 and the control years ($-\alpha_{1}^{\text{contdur}}=-0.013\pm 0.023$, p=0.26), although the mean slope is positive, consistent with our other results. Finally, neither controlling for phylogenetic relatedness, nor lockdown stringency, nor disparities in fix frequency between datasets, led to a significant change in our results (Supporting Information Appendices C and D).

### 3.1 A notable outlier

**FIGURE 4:**
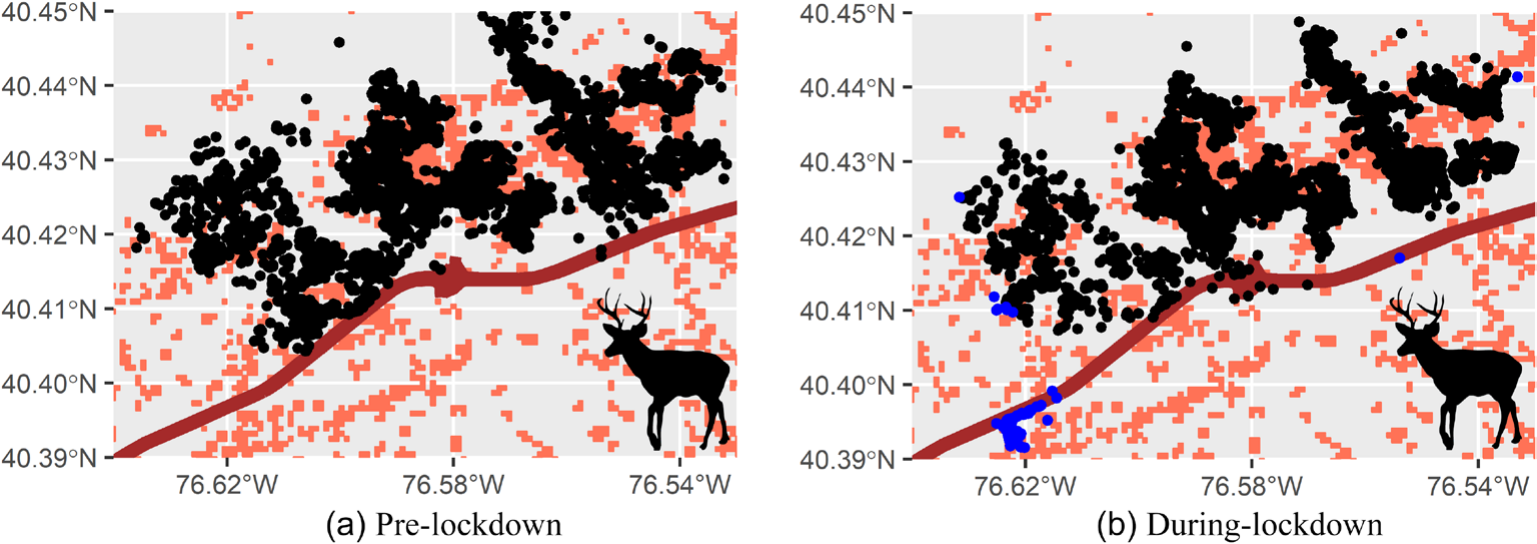
Despite being in a highly impacted area compared to most other studies (HFI = 29.7), these deer (study PD, Table 1) did not show any significant change in their response to buildings during lockdown. This can perhaps be explained by the 81 Highway (brown curve), which typically acts as a barrier to movement but became more permeable during lockdown, giving access to more open area (e.g. near the intersection in the centre and the south‐west corner). Black dots give locations within the pre‐lockdown range and blue if outside that range (as in Figure 1).

Out of the five studies with the highest HFI, four studies showed animals exhibiting significantly reduced avoidance of buildings during lockdown, while one (PD, white‐tailed deer, *Odocoileus virginianus*, in Pennsylvania) did not (Figure 2). Indeed, the mean effect size suggests a slight increase in avoidance of buildings, albeit not statistically significant. A closer look at this study revealed a study‐specific explanation for this response. The 81 Highway runs through the study site (Figure 4). Prior to lockdown, the deer remained north of this highway, suggesting this is an impenetrable barrier. During lockdown, however, some deer crossed the highway, presumably due to reduced traffic, providing them access to a broader area, including various areas without many buildings.

## 4 DISCUSSION

Our analyses revealed that terrestrial mammals living in areas with high human footprint showed reduced avoidance of buildings during COVID‐19 lockdowns. This demonstrates that human behaviour around buildings is a key driver of animal movement, not merely the built environment itself. Animals that come into regular contact with human settlements seem to have good awareness of people's behaviour, responding quickly when human mobility levels change. This finding carries important implications for designing environments conducive to human–wildlife coexistence, for example by guiding human and traffic movements in ways that minimise disturbance in key areas, like essential habitat corridors (Bastille‐Rousseau & Wittemyer, 2021; Brennan et al., 2022). Importantly, by generating parameter estimates for animal movement responses to quasi‐experimental changes in human mobility levels, our study makes a significant contribution to the development of robust predictive movement models (Gomez et al., 2025; Verzuh et al., 2026) and forecasting tools (e.g. digital twins), for simulating different disturbance and mitigation scenarios (Patchett et al., 2025; Potts & Börger, 2023; Riotte‐Lambert & Matthiopoulos, 2020). Our results resonate with an earlier single‐species study of pumas (study CP in Table 1), who relaxed their avoidance of urban areas, but not other built but less developed environments, during the lockdown (Wilmers et al., 2021).

It remains unclear at this stage why a similar reduction in the avoidance of buildings was not observed in more remote areas, as we lacked sufficient replication across species and study sites to distinguish between the effects of environmental filtering (Suraci et al., 2021) and habituation (Samia et al., 2015). In other words, it is possible that the species that live in highly impacted areas have greater behavioural plasticity than those from more remote locations (Tuomainen & Candolin, 2011). Alternatively, individuals from the same species, but living in habitats where human contact is generally lower, may find it more difficult to detect changes in human behaviour than those in higher‐HFI areas (Gaynor et al., 2024). These explanations are not mutually exclusive, though (Burton et al., 2024), and we suspect that both will apply to varying degrees across different sites. While we detected no phylogenetic signal in our analyses, investigating these issues further is arguably a priority, if we are to make progress with understanding what makes ‘winners’ and ‘losers’ in the Anthropocene (Gomez et al., 2025) and understand and predict how animals respond and adapt to increasingly changed environments (Cordes et al., 2025).

Since our study took advantage of an unexpected global event, we were constrained to using data that had originally been collected for a variety of different purposes (Rutz et al., 2020). While this is unavoidable, it is important to explore how data limitations may have affected our analyses, as well as think about how future data gathering efforts could be improved. First, there were significant sampling biases with regard to taxa, habitat composition and geographic regions. For example, a third of the studies were from the United States, but only one came from Asia and none from Oceania; over a third were from the cervid family, but there were none for primates, marsupials or rodents. Improved data coverage would no doubt enable deeper biological insights. Second, the fix frequency of GPS tags ranged from one location every 12 min to one per day (Table 1). This is not ideal, as it is known that the effect size returned by iSSA can vary with fix frequency, steadily increasing by up to a factor of two from arbitrarily high frequencies to arbitrarily low ones (Avgar et al., 2016; Moorcroft & Barnett, 2008). However, when we accounted for an effect of fix frequency, we saw almost no change in results (Supporting Information Appendix C). This indicates that disparities in fix frequency are unlikely to be having a big effect on our results. Third, and perhaps more problematic, we did not have control years for many of the studies, and even for those where baseline data were available, they came from different years (2018, 2019, 2021, 2022) and almost always different individuals. Having data for the same individuals for 2020 and 2019, and ideally, additional baseline years, would have allowed us to better control for any seasonal effect in ‘typical’ years, as well as conduct robust comparisons between equivalent times across years, rather than relying mainly on before and during the lockdown periods within 2020 (Patchett et al., 2025).

Data in a more consistent, complete format would also allow finer‐grained analyses of animal movement patterns. For example, our results pointed towards the mean lockdown effect being more pronounced amongst the carnivores we studied than the herbivores, by about a factor of two. However, despite this large average effect size, this difference was not significant, which is perhaps due to not having enough example species of each group. It is conceivable that carnivores in high‐HFI areas exhibit greater behavioural plasticity than herbivores (Suraci et al., 2021) but more data are needed to demonstrate this. Likewise, questions related to diel effects on human avoidance are relevant, as various species have been observed to avoid humans more at day than at night (Gaynor et al., 2018; Whittington et al., 2022); examining this effect would require higher frequency observations than achieved by several of our datasets (Table 1).

Although we have focused on buildings, as a general indicator of human settlements (and baseline activity) across all datasets, there were other hotspots of human disturbance. For example, during the lockdown periods, many countries and municipalities allowed people to visit large open recreational or wilderness areas, causing localised spikes of human mobility (Olejarz et al., 2023). Many of our more rural studies, therefore, are likely to have seen an increase in human mobility during lockdown. This may explain why the trend line in Figure 3c crosses the vertical axis below 0, suggesting that, in low‐HFI areas, animals actually increased their avoidance of buildings during lockdown. That said, this relationship was not significant and should therefore be interpreted cautiously.

Roads provide another example of human infrastructure which may have affected animal movement in different ways during lockdown, as compared to other times. There was generally a lot less traffic (Yasin et al., 2021), and traffic flow is likely a key reason for roads being a barrier to animal movement (Corradini et al., 2021). Indeed, it is already known that terrestrial mammals were more likely to be found closer to roads during lockdown (Tucker et al., 2023) and this is a topic of ongoing research by some of the present authors. Figure 4 illustrates how the presence of a major road can modulate the effect of buildings, driving both the fine‐grained movement decisions and emergent space use patterns of animals, which cannot be explained by considering either roads or buildings in isolation. We suggest that future research should examine these interacting effects of buildings and roads on animal movements, although the effects of roads on movement are multifarious, hence difficult to pin down: for example they can be either barriers or corridors, and can have effects that extend far beyond the width of the road itself (Benítez‐López et al., 2010; Bischof et al., 2017; Jaeger et al., 2005).

To address the lack of consistent, longitudinal, global studies on animal movement, major community efforts are underway to improve data coverage, standardisation and discovery (Davidson et al., 2025; Rutz, 2022a). Research consortia are able to couch system‐specific studies within larger, co‐ordinated data gathering efforts with agreed aims to monitor animal movement across taxa, regions and environmental contexts, and over multi‐year periods. For example, the National Geographic Society's Living Pathways programme will coordinate the tracking of animals along gradients of land modification and human disturbance worldwide—with the goal of identifying pathways towards sustainable human–wildlife coexistence in unprotected areas.

Combining such broad‐range studies with the emerging sophisticated techniques for modelling animal movement (Abrahms et al., 2021; Gomez et al., 2025; Potts & Börger, 2023; Williams, Taylor, et al., 2020) will allow us to develop a deep mechanistic understanding of how animal movement is affected by different types of human activity. This will enable the design of effective environmental planning strategies and conservation interventions, informed by the behavioural complexity and plasticity of wild animals.

## AUTHOR CONTRIBUTIONS

Jonathan R. Potts, Luca Börger and Christian Rutz conceived and designed the research, with input from Marlee A. Tucker, Scott W. Yanco, Diego Ellis‐Soto, Thomas Müller and Ruth Y. Olive. Jonathan R. Potts analysed the data, with input from Marlee A. Tucker. Jonathan R. Potts, Luca Börger, Federico Ossi and Christian Rutz co‐ordinated the project. All other authors contributed data. Jonathan R. Potts, Luca Börger and Christian Rutz wrote the manuscript. All authors gave feedback and approved the submitted version.

## CONFLICT OF INTEREST STATEMENT

CR is Founding President of the International Bio‐Logging Society (2017–2024), Chair of the COVID‐19 Bio‐Logging Initiative (2020–), Project Lead of the Urban Exploration Project (2024–), an advisor to UNEP's Convention on the Conservation of Migratory Species of Wild Animals (2015–) and Save the Elephants' Continental Tracking Initiative (2024–), and contributes to collaborative efforts to launch a global tag registry and develop the Move BON initiative.

## Supporting information


**Appendix A:** Effect of building resolution.
**Appendix B:** Details regarding the control years.
**Appendix C:** Effects of phylogeny, fix frequency, and removing control variables.
**Appendix D:** Effect of containment health index.
**Appendix E:** Alterations to the core model.
**Appendix F:** Ethical permissions.
**Table S1:** Ethical and other approvals. The first column gives the ID of the study (see Main Text Table 1), the second details the author and email (redacted for blind peer review), the third gives the ethical committee, the fourth the approval date, and the fifth is the reference number. The final three columns give details, approval date and reference number of any additional approvals given.
**Table S2:** Extents of each study site. The first column gives the ID of the study (see Main Text Table 1). The other columns are, respectively: minimum longitude, maximum longitude, minimum latitude, and maximum latitude of all the observed GPS locations used for our analysis of each study system.
**Table S3:** Details of effect sizes from Figure 2 in the Main Text. The first column gives the study ID and the second is the study name. The third and fourth are the effect sizes (mean ± SE) pre‐ and during‐lockdown, respectively. The fifth is the difference between during lock down and pre‐lock down.

## Data Availability

All data and code required for the analysis in this article are found on Zenodo, https://doi.org/10.5281/zenodo.15309885 (Potts et al., 2025).
