# Supplementary material for: Understanding mammal avoidance of human settlements
Source: J Anim Ecol. 2026 Jul 1;95(8):1504–17. doi: 10.1111/1365-2656.70298 (PMC13439596; doi:10.1111/1365-2656.70298)
Supplement: Supplementary file 1 [file JANE-95-1504-s001.pdf]

Supplementary Information for 'Understanding mammal avoidance of human settlements'

Supplementary Appendix A: Effect of building resolution

When we changed the pixel resolution for building data from 100m to 50m, we still observed an insignificant positive relationship between avoidance of buildings and HFI ($-\alpha_1^{\text{pre}} = 0.029 \pm 0.035$, $p = 0.11$; as in the Main Text, all best-fit values are reported as mean ± 95% CI). During lockdown, like with the 100m resolution, there is a negative relationship, but this time it is weaker and not statistically significant ($-\alpha_1^{\text{dur}} = -0.0057 \pm 0.030$, $p = 0.71$). The difference between pre- and during- is also weaker, but still negative ($-\alpha_1^{\text{diff}} = -0.022 \pm 0.033$, $p = 0.20$). Changing to a 150m resolution, there is a very slight, and insignificant, negative relationship between avoidance of buildings and HFI ($-\alpha_1^{\text{pre}} = 0.0021 \pm 0.025$, $p = 0.86$). During lockdown, as with the 50m resolution, there is a weaker negative relationship than for 100m ($-\alpha_1^{\text{dur}} = -0.0068 \pm 0.025$, $p = 0.59$), and the difference is also weaker but still negative ($-\alpha_1^{\text{diff}} = -0.0094 \pm 0.025$, $p = 0.46$). Overall, these results are qualitatively similar to those for our initial choice of a 100m resolution.

Supplementary Appendix B: Details regarding the control years

Here, we repeated the analysis described in the Main Text but for a control year (see Table 1 for details of which year for which study). For this, we split the data from the control year in an analogous way to the 2020 data. For example, for the WE data, the lockdown period was 13 March - 14 May and the control year was 2019. Therefore we split the data into 'period 1' from 1 Jan 2019 - 12 March 2019, and 'period 2' from 13 March 2019 - 14 May 2019.

Of the 35 studies, there were 8 for which we did not have data from a control year that were sufficient for performing step selection analysis (ZW, FC, MW, YE, SW, SZ, ME, and TL; Table 1 in Main Text). There were three further studies for which we only had data during period 1 (BA, SJ, and WS). Hence the analysis that compared period 2 with the 'during lockdown' period (in 2020) used 27 datasets in total, whereas the one comparing period 1 with period 2 (both in the control years) used 24 datasets.

Supplementary Appendix C: Effects of phylogeny, fix frequency, and removing control variables

To test whether phylogenetic relationships affect our central result (that α_1^{diff} is significantly different from 0), we used 20 mammalian phylogenetic trees sampled from those constructed by Upham et al. (2019) and free to download from `https://vertlife.org/phylosubsets/`. For each tree, we fitted a model of the form $\beta_{\text{build},i}^{\text{diff}} = \alpha_0^{\text{diff}} + \alpha_1^{\text{diff}} H_i + n_{k_i} + p_{k_i} + \epsilon_i$. Here, k_i is a number indexing the species for study i, and $n_{k_i} \sim N(0, \sigma_n^2)$ are normal i.i.d. random variables accounting for species-level differences. The vector $\mathbf{p} = (p_1, \ldots, p_m) \sim N(0, \sigma_p^2 P)$ accounts for phylogenetic correlations between species, where P is a covariance matrix given by the phylogenetic tree, σ_p^2 is a variable denoting the amount of variance due to phylogenetic effects, and m is the number of species (here, $m = 22$). We fitted this model using the `metafor` package in `R` (Viechtbauer, 2010), following the protocol of Cinar et al. (2022). In all 20 cases, we found essentially no phylogenetic effect, i.e. the unbiased estimator of σ_p^2 was $\hat{\sigma}_p^2 < 10^{-8}$. There appears to be some inter-species variation, as $\hat{\sigma}_n^2 = 0.12$, but it does not affect the central result of the paper, which is that α_1^{diff} is significantly different from 0. Indeed, the above phylogenetically-controlled model estimated α_1^{diff} as $\alpha_1^{\text{diff}} = 0.034 \pm 0.022$ ($p = 0.0025$), which is the same mean to two significant figures as the value $\alpha_1^{\text{diff}} = 0.034 \pm 0.024$ calculated without controlling for phylogenetic relationships (see Main Text).

In step selection analysis, fix frequency can alter the observed effect size (in this case $\beta_{\text{build},i}$) by up to a factor of two between arbitrarily low frequencies and arbitrarily high frequencies (Moorcroft and Barnett, 2008; Avgar et al., 2016). Whilst this is a bounded effect, which importantly cannot affect the sign of $\beta_{\text{build},i}$, it is worth checking that controlling for this does not affect the observed relationship between HFI and $\beta_{\text{build},i}^{\text{diff}}$, as we have used studies with varying fix frequencies. To this end, we fitted a model of the form $\beta_{\text{build},i}^{\text{diff}} = \alpha_0^{\text{diff}} + \alpha_1^{\text{diff}} H_i + \alpha_F F_i + \epsilon_i$, where F_i is the fix build of study i in units of hour^{-1}. The best-fit value for α_1^{diff} was $\alpha_1^{\text{diff}} = 0.034 \pm 0.025$, which compares with $\alpha_1^{\text{diff}} = 0.034 \pm 0.024$ calculated without controlling for fix build. The best-fit value for α_F is $\alpha_F = 0.050 \pm 0.160$, which is not statistically significant ($p = 0.54$).

Finally, we tested the effect of removing the three control variables: forest cover, home ranging, and elevation. The resulting model estimated $\alpha_1^{\text{diff}} = 0.036 \pm 0.025$ ($p = 0.0050$), which is very close to the value of $\alpha_1^{\text{diff}} = 0.034 \pm 0.024$ calculated with the aforementioned three control variables. Therefore these control variables are not having any significant effect on the central result.

Supplementary Appendix D: Effect of containment health index

To measure lockdown stringency, we used the Stringency Index (SI) (Hale et al., 2021), fitting a model of the form $\beta^{\text{diff}}_{\text{build},i} = \alpha^{\text{diff}}_0 + \alpha^{\text{diff}}_S S_i + \epsilon_i$, where S_i is the stringency index for study i. There was no significant relationship with the avoidance of, or attraction to, buildings either pre-lockdown ($\alpha^{\text{pre}}_S = 0.0038 \pm 0.028$, $p = 0.79$) or during-lockdown ($\alpha^{\text{dur}}_S = 0.0038 \pm 0.026$, $p = 0.77$). The difference in effect is similarly not significant ($\alpha^{\text{diff}}_S = 0.0043 \pm 0.027$, $p = 0.75$). When including both HFI and SI, in a model of the form $\beta^{\text{diff}}_{\text{build},i} = \alpha^{\text{diff}}_0 + \alpha^{\text{diff}}_1 H_i + \alpha_S S_i + \epsilon_i$, the best-fit values are $\alpha^{\text{diff}}_1 = 0.034 \pm 0.025$ and $\alpha_S = 0.0043 \pm 0.024$. In other words, the best-fit value for α_1 is almost unchanged from the situation where we do not account for SI and the effect of SI is insignificant ($p = 0.72$).

Supplementary Appendix E: Alterations to the core model

For the data on coyotes in Wisconsin (WC), we did not include turning angle distributions. The sequences where successive fixes were three hours apart tended to be very short. When including turning angles in iSSA, it is necessary to discard the first two locations in each of these sequences, whereas without turning angles only one needs to be discarded. With the WC data, incorporating turning angles would have meant discarding 80% of the datapoints.

For the datasets from Norway (ND, NM, NR) and Namibia (NC), we were unable to extract data on elevation using the same protocol as for the other datasets. Therefore we did not include elevation in analysis of these datasets. Removing these five datasets (NC, ND, NM, NR, WC) from the analysis led to similar results to keeping them in. In particular, without these five datasets, $\alpha^{\text{diff}}_1 = 0.034 \pm 0.025$ ($p = 0.009$). This is the same mean, to two significant figures, as the value $\alpha^{\text{diff}}_1 = 0.034 \pm 0.024$ calculated when including all 35 datasets (see Main Text)

Supplementary Appendix F: Ethical permissions

The COVID-19 Bio-Logging Initiative received approval for secondary data usage from the School of Biology Ethics Committee, University of St Andrews, UK (PI: C. Rutz; reference: SEC21011; date approved: 9 March 2021). For each of the 35 datasets used in the present study, data providers confirmed that:

1. The collection of the data was undertaken, accounting for the time and location of the activity:

    (a) in as humane and painless a manner as possible;

   (b) in accordance with any applicable laws;
   (c) with appropriate precautions in place to minimize interference, risk, or adverse consequences to the animals or the ecosystems of which they are part.

2. If involving endangered or CITES (I, II or III) listed species, the data collection could not be undertaken with a species not listed by CITES.

3. Any ethical approvals or favourable ethical opinions required for the original activity were obtained.

4. Any other approvals or licences required for the original activity were obtained.

Table S1 collates information submitted by data providers about approvals or licenses relevant to items (3) and (4). Author names have been redacted for peer review but will be re-instated should the manuscript be accepted for publication.

Table S1. Ethical and other approvals. The first column gives the ID of the study (see Main Text Table 1), the second details the author and email (redacted for blind peer review), the third gives the ethical committee, the fourth the approval date, and the fifth is the reference number. The final three columns give details, approval date and reference number of any additional approvals given.

ID	Author	Ethical Committee	Date	Ref No.	Other approval	Date	Ref No.
AD AW		Amt der Niederosterreichischen Landesregierung	05/07/2018	LF1-TVG-59/001-2018	NA	NA	NA
BA		NA	NA	SISBIO- 53798-17	NA	NA	NA
BW		Comissão de Ética no Uso de Animais, Faculdade de Medicina Veterinária, Universidade de São Paulo	09/06/2022	8983240322	ICMBio/ SISBIO	04/04/2019<br>23/04/2020<br>06/01/2021<br>04/02/2022<br>06/04/2023	61097-3<br>61097-4<br>61097-7<br>61097-8<br>61097-9
CD		Wildlife Committee Autonomous Province of Trento	20/09/2011<br>23/04/2015<br>18/09/2018	NA	NA	NA	NA
CP		UCSC IACUC	16/01/2020	Wilmc1912	NA	NA	NA
CW		UM IACUC	NA	AUP 066-21	Parks Canada research permits, Alberta Environment and Parks Research and Collection permits	NA	NA
DD		Norwegian Experimental Animal Board	biannual	FOTS ID15947	Norwegian Directorate for Nature Management	NA	2014/14415

ID	Author	Ethical Committee	Date	Ref No.	Other approval	Date	Ref No.
FC		NA	NA	NA	Prefet de la region Ile de France	19/02/2019	IDF-2019-02-19-003
ID		SIU IACUC	20/09/2021	21-028	NA	NA	NA
KL		Kenya Wildlife Service	NA	KWS/904	WRTI Research permit	NA	WRTI-0452-08-24
ME		Animal Care and Use Committee, University of Idaho	06/10/2015	2015-39	Gorongosa National Park, Department of Scientific Services	NA	PNG/DSCi/C92/2018
MW		Institutional Animal Care and Use Committees of Mississippi State University	NA	09-004<br>12-012<br>15-013<br>17-119	State University of New York College of Environmental Science and Forestry	NA	180505
NC		Leibniz IZW	01/01/2020<br>01/01/2024	NA	Ministry of Environment Namibia	yearly	NA
ND		Norwegian Animal Research Unit	NA	FOTS ID 26077<br>FOTS ID 29945<br>FOTS ID 30049	Norwegian Environmental Agency		
PD<br>NY		NA	NA	NA	New York Department of Environmental Conservation	28/10/2021	LCPSCI-2100
SJ		NA	NA	NA	Ministry of Republic of Slovenia for Environment and Spatial planning	30/01/2018	35601-115/2017-4

ID	Author	Ethical Committee	Date	Ref No.	Other approval	Date	Ref No.
SL		Slovenian Environmental Agency	07/07/2020	35601-76/2020-6	Ministry of Natural Resources and Spatial Planning	04/03/2024	35606-19/ 2024-2560-6
SW/SZ		Ezemvelo Permit	NA	E/5130/02	NA	NA	NA
TL TW		Kafkas University ethical committee (KAÜ HADYEK)	03/04/2024	KAÜ-HADYEK/ 2024-071	Türkiye's Department of Nature Conservation and National Parks and the the Ministry of Agriculture and Forestry	08/07/2024	E-21264211-288.04-1602322, 72784983-488.04-114100
WB/WY		UW IACUC	NA	4381-01	WDFW	NA	SCP 21-066
WC WF		Univ of Wisconsin-Madison College of Agriculture and Life Sciences Animal Care Use Committee	29/09/2024	MS2_A005905-R02-A01	Wisconsin Department of Natural Resources Scientific Research License	10/12/2024	SRLR-24-01
WE		Wyoming Chapter 33	from 2016	394	NA	NA	NA
YE		IACUC of the Smithsonian Institution	NA	17-30	NA	NA	NA
ZD		Ethics Committee of the Ministry of the Environment of the Czech Republic	03/03/2019	MZP/2019/630/361	NA	NA	NA

Table S2. Extents of each study site. The first column gives the ID of the study (see Main Text Table 1). The other columns are, respectively: minimum longitude, maximum longitude, minimum latitude, and maximum latitude of all the observed GPS locations used for our analysis of each study system.

ID	Min long	Max long	Min lat	Max lat
AD	15.2683	15.3995	48.6286	48.6740
BA	-53.6169	-53.4563	-21.6698	-21.0463
BW	-46.9914	-46.9239	-21.6105	-21.5607
CD	11.1365	11.2701	46.1058	46.1858
CP	-122.2356	-121.7191	36.9549	37.1468
CW	-116.1588	-115.0904	51.0820	52.0451
DD	8.8817	10.5194	56.1924	56.4403
FC	1.9199	1.9958	42.6499	42.6940
ID	-89.1924	-88.5378	37.6013	39.5884
KL	36.7840	36.9757	-0.0599	0.1805
ME	34.1277	34.5788	-19.0711	-18.6144
MW	-91.3684	-84.6847	43.9941	46.4673
NC	16.0584	18.8000	-23.0430	-21.3678
ND	7.0654	9.4262	60.9858	62.8652
NM	10.5934	12.1299	63.0896	65.7154
NR	6.8459	11.1671	59.7876	62.1054
NY	-74.2122	-74.1700	40.5512	40.6447
PD	-76.6251	-76.5317	40.3916	40.4683
SE	27.7040	27.9363	-24.4217	-24.1570
SJ	13.4844	14.9937	44.9272	45.8914
SL	14.0861	15.2201	45.2424	45.9458
SW	31.7133	31.9931	-28.3011	-28.1384
SZ	31.7013	32.1462	-28.2931	-28.0325
TL	42.4355	42.6775	40.1751	40.3389
TW	42.1712	42.7238	40.0327	40.3479
WB	-120.6144	-117.3814	47.9809	48.9216
WC	-90.1278	-89.8881	43.3581	43.5472
WD	-111.1849	-110.0307	41.4706	42.9473
WE	-110.7445	-108.8320	43.4938	44.6158
WF	-89.5240	-89.4113	43.0452	43.1309
WS	-110.8068	-109.3698	43.3092	44.5073
WY	-120.3319	-117.2866	48.0941	48.6474
YE	94.3509	98.9723	11.1694	17.2117
ZD	13.6189	14.0923	48.8248	48.9912
ZW	13.0724	13.2377	50.1673	50.3263

Table S3. Details of effect sizes from Figure 2 in the Main Text. The first column gives the study ID and the second is the study name. The third and fourth are the effect sizes (mean ± SE) pre- and during-lockdown, respectively. The fifth is the difference between during lockdown and pre-lockdown.

ID	Name	Pre-lockdown	During lockdown	Difference
NR	Reindeer Norway	0.33 ± 1.07	-10.11 ± 9.23	-10.44 ± 9.29
NC	Cheetah Namibia	-13.31 ± 568.15	0.72 ± 1.16	14.03 ± 568.15
FC	Chamois France	0.26 ± 0.68	1.05 ± 1.10	0.79 ± 1.30
WB	Bobcat Washington	-0.90 ± 0.42	-1.59 ± 2.46	-0.69 ± 2.50
ZW	Grey wolf Czech Republic	0.37 ± 1.09	0.61 ± 1.18	0.24 ± 1.61
WS	Bighorn sheep Wyoming	-0.31 ± 0.10	-0.12 ± 0.09	0.19 ± 0.14
KL	Lion Kenya	-3.51 ± 8.22	-0.32 ± 1.08	3.19 ± 8.29
MW	Grey wolf Michigan	-1.38 ± 0.46	-1.52 ± 0.52	-0.14 ± 0.69
ZD	Red deer Czech Republic	-1.94 ± 0.46	-1.35 ± 0.64	0.59 ± 0.79
YE	Asian elephant Myanmar	-0.40 ± 0.12	-0.41 ± 0.13	-0.01 ± 0.18
SE	African elephant South Africa	0.24 ± 0.08	0.09 ± 0.10	-0.14 ± 0.12
CW	Grey wolf Canada	-2.83 ± 0.58	-2.18 ± 0.72	0.65 ± 0.93
SW	Blue wildebeest South Africa	-0.63 ± 0.20	-2.03 ± 0.40	-1.40 ± 0.44
WY	Coyote Washington	-0.15 ± 0.28	-0.48 ± 0.30	-0.33 ± 0.41
WD	Mule deer Wyoming	-0.19 ± 0.20	-0.51 ± 0.17	-0.32 ± 0.26
SZ	Plains zebra South Africa	-0.60 ± 0.18	-0.33 ± 0.27	0.27 ± 0.32
SL	Eurasian Lynx Slovenia/Croatia	-0.70 ± 0.72	-0.27 ± 0.49	0.43 ± 0.87
ME	African elephant Mozambique	-2.20 ± 1.46	0.65 ± 0.39	2.84 ± 1.51
ND	Red deer Norway	0.43 ± 0.35	0.11 ± 0.17	-0.32 ± 0.39
NM	Moose Norway	-0.63 ± 0.12	-0.82 ± 0.10	-0.19 ± 0.16
TL	Eurasian Lynx Turkey	-0.30 ± 1.04	0.22 ± 1.76	0.53 ± 2.05
AD	Red deer Austria	-0.47 ± 1.04	-0.62 ± 1.06	-0.16 ± 1.49
TW	Grey wolf Turkey	-0.29 ± 0.53	-0.50 ± 0.61	-0.20 ± 0.81
BA	Giant anteater Brazil	-0.56 ± 0.22	0.00 ± 0.31	0.56 ± 0.38
DD	Red deer Denmark	-1.44 ± 0.40	-1.13 ± 0.18	0.31 ± 0.44
WE	Elk Wyoming	-2.47 ± 0.46	-1.44 ± 0.18	1.02 ± 0.49
ID	White-tailed deer Illinois	-0.33 ± 0.16	-0.41 ± 0.16	-0.07 ± 0.23
WC	Coyote Wisconsin	0.35 ± 0.46	-0.39 ± 0.49	-0.74 ± 0.67
SJ	Golden jackal Slovenia	-0.55 ± 0.25	0.10 ± 0.28	0.65 ± 0.38
CP	Puma California	-0.51 ± 0.09	-0.39 ± 0.32	0.12 ± 0.33
BW	Maned wolf Brazil	-2.01 ± 0.72	-0.19 ± 0.28	1.83 ± 0.77
CD	Roe deer Italy	-1.18 ± 0.41	-0.23 ± 0.22	0.95 ± 0.47
PD	White-tailed deer Pennsylvania	-0.46 ± 0.11	-0.63 ± 0.11	-0.17 ± 0.16
NY	White-tailed deer New York	-0.74 ± 0.32	0.07 ± 0.06	0.82 ± 0.32
WF	Red fox Wisconsin	-0.89 ± 0.25	0.72 ± 0.33	1.61 ± 0.41

References

Avgar, T., J. R. Potts, M. A. Lewis, and M. S. Boyce. 2016. Integrated step selection analysis: bridging the gap between resource selection and animal movement. Methods in Ecology and Evolution, **7**:619–630.

Cinar, O., S. Nakagawa, and W. Viechtbauer. 2022. Phylogenetic multilevel meta-analysis: A simulation study on the importance of modelling the phylogeny. Methods in Ecology and Evolution, **13**:383–395.

Hale, T., N. Angrist, R. Goldszmidt, B. Kira, A. Petherick, T. Phillips, S. Webster, E. Cameron-Blake, L. Hallas, S. Majumdar, et al. 2021. A global panel database of pandemic policies (Oxford COVID-19 Government Response Tracker). Nature Human Behaviour, **5**:529–538.

Moorcroft, P. and A. Barnett. 2008. Mechanistic home range models and resource selection analysis: a reconciliation and unification. Ecology, **89**:1112–1119.

Upham, N. S., J. A. Esselstyn, and W. Jetz. 2019. Inferring the mammal tree: species-level sets of phylogenies for questions in ecology, evolution, and conservation. PLoS Biology, **17**:e3000494.

Viechtbauer, W. 2010. Conducting meta-analyses in R with the metafor package. Journal of Statistical Software, **36**:1–48.
